# Calmodulin interacts with Rab3D and modulates osteoclastic bone resorption

**DOI:** 10.1038/srep37963

**Published:** 2016-11-29

**Authors:** Sipin Zhu, Shek Man Chim, Taksum Cheng, Estabelle Ang, Benjamin Ng, Baysie Lim, Kai Chen, Heng Qiu, Jennifer Tickner, Huazi Xu, Nathan Pavlos, Jiake Xu

**Affiliations:** 1 Department of Orthopaedics, The Second Affiliated Hospital, Wenzhou Medical University, Wenzhou, Zhejiang, 325027 China; 2 School of Pathology and Laboratory Medicine, The University of Western Australia, Perth, WA 6009, Australia; 3 Centre for Orthopaedic Research, School of Surgery, The University of Western Australia, Perth, WA 6009, Australia; 4 School of Dentistry, Oral Biology Research Laboratory, The University of Western Australia, Perth, WA 6009, Australia

## Abstract

Calmodulin is a highly versatile protein that regulates intracellular calcium homeostasis and is involved in a variety of cellular functions including cardiac excitability, synaptic plasticity and signaling transduction. During osteoclastic bone resorption, calmodulin has been reported to concentrate at the ruffled border membrane of osteoclasts where it is thought to modulate bone resorption activity in response to calcium. Here we report an interaction between calmodulin and Rab3D, a small exocytic GTPase and established regulator osteoclastic bone resorption. Using yeast two-hybrid screening together with a series of protein-protein interaction studies, we show that calmodulin interacts with Rab3D in a calcium dependent manner. Consistently, expression of a calcium insensitive form of calmodulin (i.e. CaM1234) perturbs calmodulin-Rab3D interaction as monitored by bioluminescence resonance energy transfer (BRET) assays. In osteoclasts, calmodulin and Rab3D are constitutively co-expressed during RANKL-induced osteoclast differentiation, co-occupy plasma membrane fractions by differential gradient sedimentation assay and colocalise in the ruffled border as revealed by confocal microscopy. Further, functional blockade of calmodulin-Rab3D interaction by calmidazolium chloride coincides with an attenuation of osteoclastic bone resorption. Our data imply that calmodulin- Rab3D interaction is required for efficient bone resorption by osteoclasts *in vitro*.

Calmodulin is a versatile protein that regulates Ca(2+) homeostasis^1^, synaptic plasticity^2^, and cardiac excitability^3^. It has been implicated in osteoclast differentiation, function, and survival. Calmodulin regulates Ca2+/calmodulin-dependent kinase II (CaMKII) and Ca2+/calmodulin-dependent protein phosphatase, calcineurin^4^; both are critical for osteoclast differentiation^5^,^6^,^7^. It also mediates osteoclast survival through a mechanism involving the binding of calmodulin with the death receptor Fas^8^,^9^. In addition, calmodulin modulates acid transport and bone resorption^10^,^11^,^12^. However, the underlining molecular events by which calmodulin regulates bone resorption remain to be elucidated.

Bone resorption is a highly regulated process that requires intense vesicular trafficking to sustain the structural and functional polarity of osteoclasts and enable efficient delivery of osteolytic cargo contents (i.e. Cathepsin K) and membrane exchange between the ruffled border and the opposing basolateral surface. Vesicular trafficking is modulated by sets of genetically conserved proteins among which members of the small Rab GTPase family are now firmly established regulators^13^. To date, several Rab proteins have been implicated in bone resorption^14^, although only Rab7^15^ and Rab3D^16^ and been functionally characterized in osteoclasts. Rab7 is involved in the late endocytic pathway in the osteoclast polarization and bone resorption^15^, whereas Rab3D modulates a post-TGN trafficking step that is required for maintenance of the osteoclastic ruffled border membrane^16^ and utilizes the dynein motor complex and microtubules via its direct interaction with Tctex-1 to facilitate vesicle delivery and/or retrieval during bone resorption^17^.

Here, we identify calmodulin as a specific Rab3D interacting molecule by yeast two hybrid screening. We show that inhibition of calmodulin calcium binding perturbs this association *in vivo* by bioluminescence resonance energy transfer (BRET). Disruption of calmodulin-Rab3D interaction attenuated osteoclastic bone resorption *in vitro*. We propose that calmodulin, via modulating calcium, imparts an additional layer of regulation on Rab3D trafficking during osteoclastic bone resorption.

## Results

### Calmodulin interacts with Rab3D

**Figure 1:**
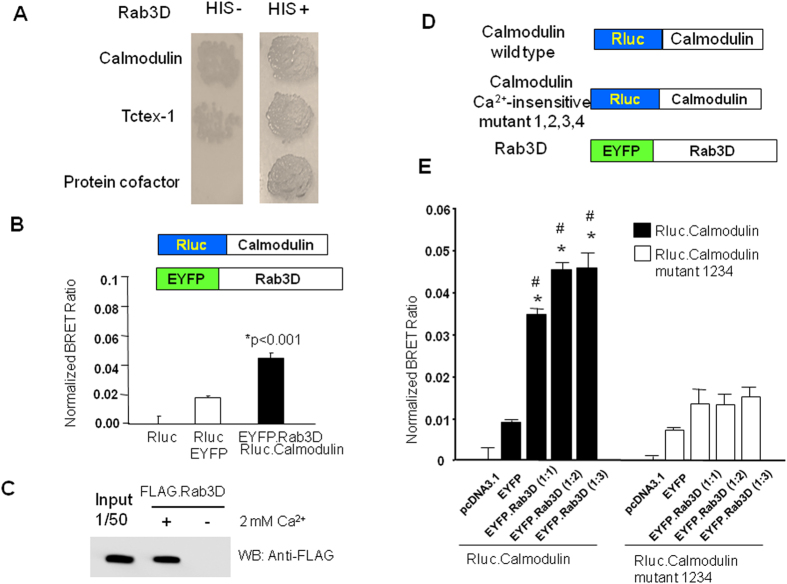
Calmodulin interacts with Rab3D. (**A**) A yeast two hybrid assay showing that Calmodulin interacts with Rab3D, by using histidine-deficient plate. (**B**) BRET assays showing that co-transfection of Rluc-Camodulin and EYFP-Rab3D fusion protein constructs resulted in a significant BRET signal. Co-expression of Rluc and EYFP is shown as a negative control. (**C**) Flag-Rab3D proteins expressed in COS cells interact with calmodulin saphorose in the presence of 2 mM calcium. *Indicates p Value < 0.001 when compared with EYFP and Rluc. (**D**) Calmodulin calcium-insensitive mutant perturbs its interaction with Rab3D. Generation of a Rluc-calmodulin construct in which four aspartic acid residues at position 23, 59, 96, 132 were substituted with alanine, mimicking a calcium insensitive form of calmodulin. (**E**) BRET assays showing that the calcium insensitive form of camodulin failed to interact with Rab3D. 1:1, 1:2 and 1:3 indicate that transfected plasmid ratio of EYFP-Rab3D/ Rluc-camodulin or EYFP-Rab3D/ Rluc-calmodulin mutant 1234. Symbol *indicates p Value < 0.001 when compared with EYFP and Rluc-camodulin control. Symbol # indicates p Value < 0.001 when compared Rluc-camodulin with Rluc-calmodulin mutant 1234.

We have previously established a yeast two-hybrid approach to successfully uncover novel Rab3D interacting partners such as Tctex-1^17^. Here we identify calmodulin as an additional binding partner of Rab3D. The interaction of calmodulin with Rab3D was verified by a yeast two hybrid assay, using a histidine-deficient plate (Fig. 1A). To further examine the interaction of calmodulin and Rab3D, we generated Rluc-camodulin and EYFP-Rab3D fusion protein constructs and performed BRET protein-protein interaction assays. As shown in Fig. 1B, co-expression of Rluc-calmodulin and EYFP-Rab3D resulted in a significant BRET signal when compared to the co-expression of Rluc and EYFP. To further confirm the interaction, an *in vitro* calmodulin sepharose-pull down assay was performed. Rab3D was cloned into a mammalian expression vector with an N terminal Flag-tagged (Fig. 1C). Flag-Rab3D proteins were prepared from COS cells transfected with pcDNA3.1-Flag-Rab3D expressing plasmids. COS cell lysates were harvested and subjected to immobilized calmodulin sepharose in the presence or absence of 2 mM calcium. As shown in Fig. 1C, Flag-Rab3D proteins bound immobilized calmodulin saphorose in the presence (but not in the absence) of calcium, indicative of a calcium dependent binding dependency.

### Calmodulin calcium insensitive mutant perturbs its interaction with Rab3D

Considering that calmodulin has four calcium binding sites via four aspartic acid residues^18^ and acts as a calcium modulator in the calcium sensitive regulation of many cellular processes, we next examined if calcium binding site of calmodulin is required for the interaction of calmodulin with Rab3D. For this, we generated a Rluc-calmodulin construct in which four aspartic acid residues at position 23, 59, 96, 132 were substituted with alanine, mimicking a calcium insensitive form of calmodulin^18^ (Fig. 1D). BRET assay results showed that the calcium insensitive form of camodulin attenuated the interaction with Rab3D (Fig. 1E).

### The preferential interaction between Calmodulin and Rab3D in its GTP-bound conformation

**Figure 2:**
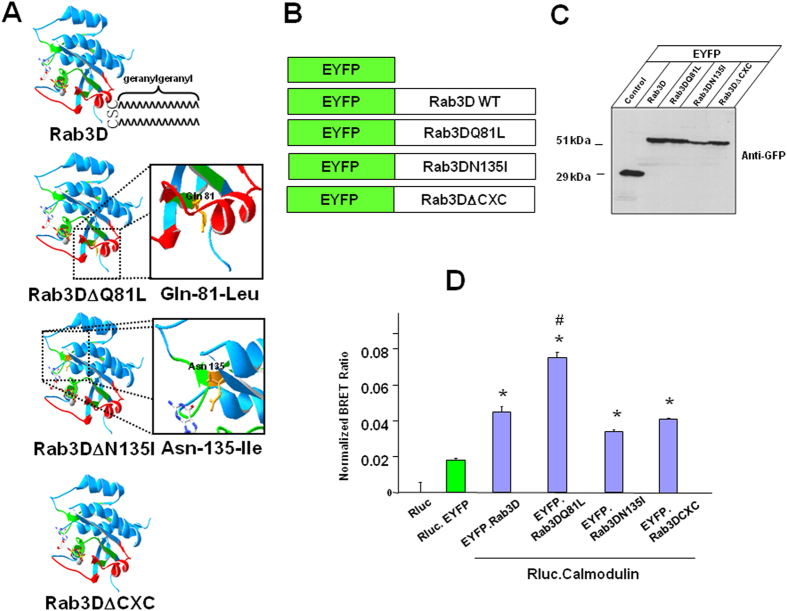
The interaction of Calmodulin with Rab3D has a closer proximity when Rab3D is GTP-bound. (**A**) Predicted molecular structures of wild-type Rab3D, GTP-bound Rab3D (Rab3DQ81L), nucleotide empty RAB3D (Rab3DN135I) and prenylation motif deletion of Rab3D (Rab3DΔCXC). (**B**) EYFP fusion protein constructs of EYFP-Rab3D, EYFP-Rab3DQ81L, EYFP-Rab3DN135I and EYFP-Rab3DΔCXC that were used for BRET assays. (**C**) Western blot analysis showing the expression of EYFP-Rab3D, EYFP-Rab3DQ81L, EYFP-Rab3DN135I and EYFP-Rab3DΔCXC proteins by anti-GFP. (**D**) BRET assays showing that calmodulin exhibited an enhanced association with a GTP-bound Rab3D (Rab3DQ81L) when compared to wild-type Rab3D, nucleotide empty RAB3D (Rab3DN135I) and prenylation motif deletion of Rab3D (Rab3DΔCXC) in BRET assays. *Indicates p Value < 0.001 when compared with EYFP and Rluc. # indicates p Value < 0.05 when compared to wild-type Rab3D, nucleotide-empty (Rab3DN135I) and prenylation motif deletion of Rab3D (Rab3DΔCXC).

Rab GTPases embed in organelle membranes via C-terminal prenylation moties where they function as molecular switches that oscillate between GTP “active” and GDP “inactive” conformations. In their active state, Rabs recruit GTP-dependent effector proteins through which they elicit their biological function at various stages of vesicular transport. Therefore, we next asked whether the interaction between Calmodulin and Rab3D was dependent on the nucleotide and/or prenylation status of Rab3D. To access this, we employed several well characterised Rab3D variants^16^, which selectively disrupt the GDP/GTP exchange i.e. GTP-bound Rab3D (Rab3DQ81L), nucleotide empty RAB3D (Rab3DN135I) and prenylation motif deletion of Rab3D (Rab3D CXC) compared to wildtype Rab3D (Fig. 2A,B). These constructs were successfully expressed as EYFP fusion proteins in transfected COS cells as confirmed by western blot analyses (Fig. 2C). As with other bona fide Rab effector protein, calmodulin exhibited a preferential association with Rab3D when locked in is GTP-bound form (Rab3DQ81L) when compared to wild-type Rab3D, nucleotide-empty (Rab3DN135I) and prenylation motif deletion of Rab3D (Rab3D CXC) in BRET assays (Fig. 2D). These data imply that the interaction of calmodulin with Rab3D is largely influenced by its active GTP-bound state.

### Calmodulin and Rab3D are co-expressed during osteoclast formation and co-sediment in membrane fractions of osteoclasts

**Figure 3:**
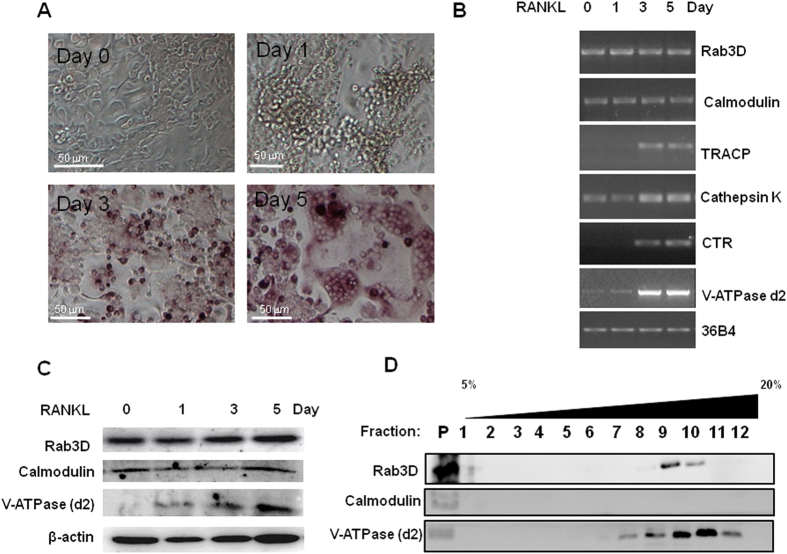
Calmodulin and Rab3D are co-expressed during osteoclast formation and co-fractionated in the membrane fraction of osteoclasts. (**A**) Representative images from BMMs cultured in the present of M-CSF and RANKL for a period of 0, 1, 3, 5 days and then fixed and stained for TRACP activity. (**B**) Semi-quantitative RT-PCR showing the co-expression of calmodulin and Rab3D along with osteoclast marker gene expression of TRACP, NFATc1, DC-STAMP, V-ATPase d2, and calcitonin receptor. (**C**) Western blot analysis showing that calmodulin and Rab3D proteins are expressed at similar kinetics during osteoclastogenesis. (**D**) Sucrose gradient sedimentation assays showing that Rab3D and calmodulin co-fractionate in the large membrane faction (P = Pellet). Note that all Western blot results with cropped blots are displayed from well established sizes of proteins; calmodulin, Rab3D and VATPase (d2).

To begin to probe the potential relevance of the calmodulin-Rab3D association in osteoclasts, we first compared the gene expression profile of calmodulin and Rab3D in osteoclasts and their precursor cells. Bone marrow macrophages (BMM) were cultured in the present of macrophage colony stimulating factor (M-CSF) and receptor activator of NF-κB ligand (RANKL) for a period of 0, 1, 3, 5 days and then fixed and stained for tartrate resistant acid phosphatase (TRACP) activity, showing the presence of multinucleated TRACP positive osteoclast like cells (Fig. 3A). In a parallel experiment, semi-quantitative RT-PCR was performed. Calmodulin gene expression appears to be constitutive during osteoclastogenesis by RANKL at an expression kinetic similar to that of Rab3D (Fig. 3B). Osteoclast marker gene expression of TRACP, Cathepsin K, V-ATPase d2, and calcitonin receptor (CTR) were induced by RANKL as compared to 36B4 gene expression as an internal control (Fig. 3B). Further, Western blot analysis showed that Calmodulin protein is constitutively expressed during osteoclastogenesis similar to Rab3D protein expression (Fig. 3C).

**Figure 4:**
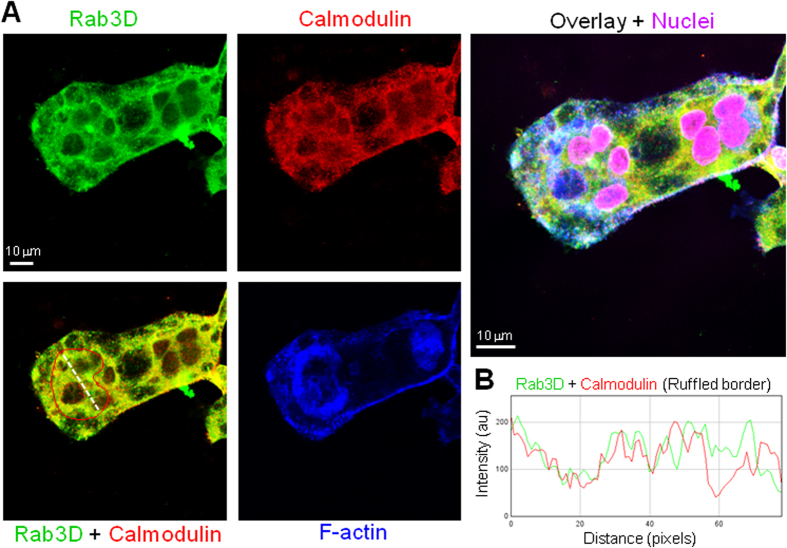
Colocalisation between Rab3D and calmodulin in bone-resorbing BMM-derived osteoclasts. (**A**) Representative confocal microscopy images of individual and overlay fluorescent channels of Rab3D (green), calmodulin (red), F-actin (blue) and nuclei (magenta). Colocalisation between Rab3D and calmodulin appears as yellow in overlay. Red line demarcates the ruffled border region within the sealing zone. White line corresponds to the correlative linescan analysis in (**B**). Bar = 10 μm.

Next, sucrose gradient sedimentation assays were performed to examine calmodulin and Rab3D co-fractionation. Rab3D is present in small vesicles (F9-10) and large plasma membrane fractions (P) (Fig. 3D). Interestingly, calmodulin co-fractionated with Rab3D but only in the large membrane faction (P) (Fig. 3D). By comparison, V-ATPase (d2) was also present in both vesicle and membrane fractions (Fig. 3D). Moreover, an association between Rab3D and calmodulin was further confirmed in bone-resorbing osteoclasts by immunofluorescence confocal microscopy (Fig. 4). In this instance colocalisation (yellow colour) was observed upon overlay of individual fluorescent channels for Rab3D (green) and Calmodulin (red), which were detected using antibodies specific to Rab3D and calmodulin, as validated in the Western blot analysis above (i.e. Fig. 3C). Interestingly, a subset of Rab3D-calmodulin colocalisation was observed within the F-actin ring/sealing zone (blue) that typically denotes the ruffled border membrane (Fig. 4A, region circumscribed by red line). This colocalisation was confirmed by correlative linescan analysis (Fig. 4B) which revealed overlap between the fluorescent peaks of Rab3D and calmodulin within the ruffled border region (Fig. 4A, white dashed line).

### Functional blockade of the calmodulin-Rab3D interaction by calmidazolium chloride attenuates osteoclastic bone resorption

**Figure 5:**
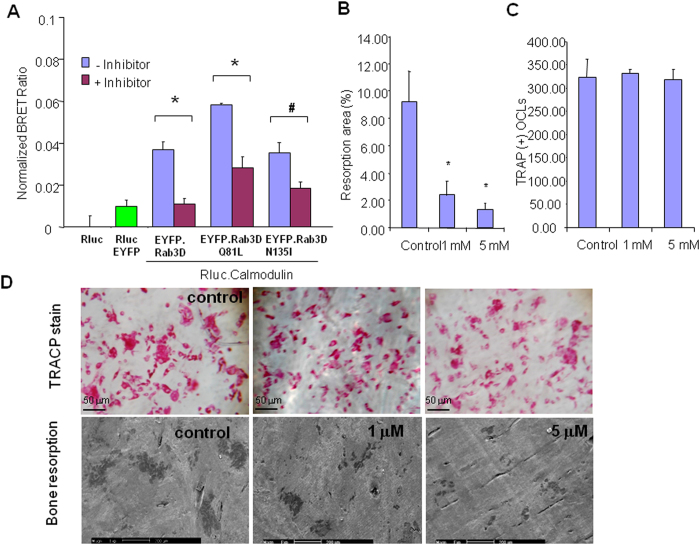
Functional blockade of the interaction of Rab3D and calmodulin by calmidazolium chloride attenuates osteoclastic bone resorption. (**A**) BRET assays showing the effect of calmidazolium chloride on the interaction of calmodulin and Rab3D, Rab3DQ81L, Rab3DN135I and Rab3DΔCXC; respectively. (**B**) Treatment of osteoclasts with calmidazolium chloride inhibits osteoclastic bone resorption with quantitative analysis of bone resorption areas. BMM derived osteoclasts were seeded into bone slices in the presence and absence of calmidazolium chloride for 24 hours. (**C**) Total number of TRACP positive osteoclastic like cells. (**D**) Representative images of bone resorption assays showing the effect of calmidazolium on TRACP positive osteoclast morphology (upper panel), and osteoclastic bone resorption SEM images (lower panel). Scale bars are shown. * and ^#^Indicate p Value < 0.001, and < 0.05 respectively when compared to untreated control.

Finally, we set out to define the impact of the interaction of calmodulin with Rab3D on osteoclast function. To this end, we first tested the effect of calmidazolium chloride on the interaction of calmodulin and Rab3D using BRET assays. Interestingly, calmidazolium chloride perturbs the association of calmodulin and Rab3D, Rab3DQ81L, Rab3DN135I and Rab3DΔCXC (Fig. 5A). Further, to examine the effect of calmidazolium on osteoclastic bone resorption, BMM derived osteoclasts were seeded into bone slices in the presence and absence of calmidazolium chloride for 24 hours. Treatment of osteoclasts with calmidazolium chloride inhibited osteoclastic bone resorption (Fig. 5B) but did not affect the total number (Fig. 5C) and morphology of TRACP positive osteoclastic like cells at 1 μM, and 5 μM (Fig. 5D). Taken together, these data suggest that treatment of calmidazolium chloride perturbs the interaction of calmodulin with Rab3D, an effect that coincides with the attenuation of osteoclastic bone resorption *in vitro*.

## Discussion

**Figure 6:**
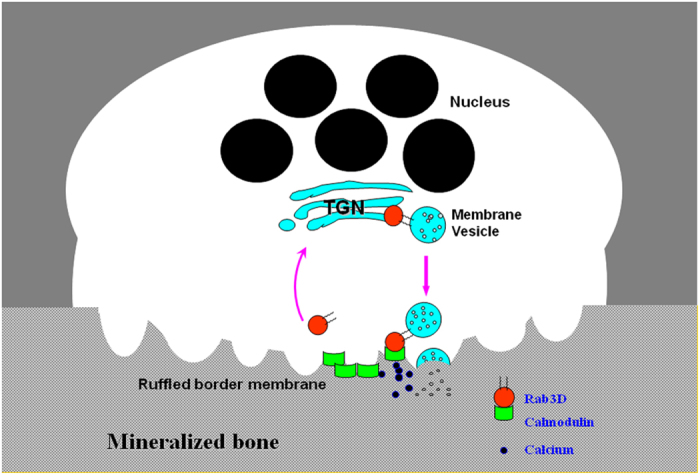
A working model illustrating that calmodulin is concentrated on the ruffled border membrane in osteoclasts and mediates bone resorption process^8^. Rab3D interaction with calmodulin is implicated for a role in bone resorption when Rab3D-bearing vesicles reach the resorbing ruffled border compartment of an osteoclast.

Calmodulin has versatile roles in regulating intracellular calcium homeostasis and diverse cellular processes including osteoclastic bone resorption. In this study we document that calmodulin interacts with Rab3D in a calcium-dependent manner. Both calmodulin and Rab3D proteins co-occupy membranes factions by a sucrose gradient ultracentrifugation sedimentation assay and colocalise in the ruffled border by confocal microscopy. Functional blockade of calmodulin and Rab3D interaction by calmidazolium chloride resulted in an attenuation of osteoclastic bone resorption. Considering that calmodulin is concentrated on the ruffled border membrane in osteoclasts^8^, and that Rab3D is a functional requirement for ruffled border maintenance^17^, it is plausible that calmodulin, through its interaction with Rab3D facilitates the delivery and/or calcium sensitively of Rab3D-bearing vesicles at the ruffled border membrane during bone resorption (Fig. 6).

Bone resorption by osteoclasts is a multi-step process which culminates in the removal of an inorganic mineral layer primarily composed of cystralline hydroxylapatite and subsequent degradation of the underlying organic phases. This process involves continual content delivery and membrane recycling through vesicle trafficking, governed largely small Rab GTPases. Rab proteins have been implicated in the regulation of distinct events in vesicle transport on the exocytotic, endocytotic and transcytotic pathways^19^,^20^. Because of this, in recent years, there has been mounting interest surrounding the role of Rab3D in intracellular transport. Rab3D was shown to mediate exocytosis in mast cells^21^, adipocytes^22^, and acini cells^23^. GTPase- deficient Rab3D decreased nutrient induced insulin release^24^. Moreover, actin coating of secretory granules during regulated exocytosis has been shown to correlate with the release of Rab3D^25^. In addition, Rab3D has been suggested to play a role in the regulation of apically directed transcytosis in rat hepatocytes^26^ and appears to be essential for apical transport in polarized epithelia^27^. Our previous data indicates that Rab3D modulates a post-TGN trafficking step that is required for osteoclastic bone resorption^16^. Furthermore, we have shown that Rab3D interacts with Tctex-1 which regulates microtubule-directed trafficking of Rab3D vesicles via cytoplasmic dynein^17^. The present study further extends the role of Rab3D in bone resorption by binding to calmodulin; a molecule previously implicated in acid secretion and bone resorption^10^,^11^,^12^.

During the formation of ruffled border membrane domains, the osteoclastic plasmalemma can be further divided into several specialised functional domains. These include the basolateral domain and a functional secretory domain^28^,^29^. In fact more recent evidence suggests that the ruffled border is not a continuous domain but rather segregated into an “uptake” and “secretory” domain reflecting its dynamic endo-exocytic intracellular trafficking routes^30^. Therefore it is likely that Rab3D mediates bone resorption along the exocytic pathway through its direct interaction with calmodulin that is located in the ruffled border membranes. In other systems, calmodulin has been shown to stimulate GTP binding to Rab3A that is complexed with GDI, which leads to the formation of an active GTP-bound form of the Rab3A-Ca (2+)/Calmodulin complex in synaptic membranes of activated nerve termini^31^. Similarly, interaction between Rab3B and calmodulin was Ca (2+)-dependent^32^. These findings provide evidence that Rab3B is primarily localized with the particulate fraction and that Ca (2+)/calmodulin could regulate function of this GTPase in platelets^32^. It remains to be elucidated whether other Rab proteins might also interact with calmodulin and facilitate these molecular pathways in osteoclasts and other cells.

Previously, structural analysis revealed four Ca (2+)-binding domains in calmodulin that are important for the function of calmodulin, together with hydrophobic regions represent the sites of interaction with pharmacological agents^33^. The three-dimensional structure of calmodulin has been determined crystallographically at 3.0 A resolution. The molecule consists of two globular lobes connected by a long exposed alpha-helix, and each lobe is able to bind two calcium ions through helix-loop-helix domains^34^. The flexibility of the protein may explain the fact that calmodulin is able to bind many different targets^35^. We have found that the interaction of calmodulin with Rab3D is calcium dependent. During osteoclastic bone resorption, free calcium is to be released in the resorption compartment, which could in turn further facilitate the interaction of calmodulin with Rab3D. It has been suggested that a Rab3-calmodulin complex generated by elevated Ca (2+) concentrations mediated at least some of the effects of the GTPase and limited the number of exocytic events that occurred in response to secretory stimuli^36^. We propose that the recruitment of calmodulin by Rab3D is an important requirement for osteoclast-mediated bone resorption.

Identification of interacting partners to Rab proteins will be important for drug design, for instance, Plekhm1 was found to co-localize with Rab7 to late endosomal/lysosomal vesicles, a putative function in vesicular transport in the osteoclast. This has been implicated in the development of osteopetrosis^37^. Interestingly, alendronate inactivates osteoclasts by mechanisms that impair their intracellular vesicle transport, with apoptosis being only a secondary phenomenon to this^38^. The anti-resorptive activity of NE10790 is thus likely due to disruption of Rab-dependent intracellular membrane trafficking in osteoclasts^39^. Given that Rab-dependent intracellular membrane trafficking in osteoclastic bone resorption has been proposed to be a target of nitrogen-containing bisphosphonate drug NE10790^39^, defining Rab3D interacting partners might facilitate the design of the next generation of bisphosphonate drugs.

## Materials and Methods

### Two-hybrid screening

Mouse Rab3D cDNA was inserted into pBTM116 and used to screen a pVP16-based yeast two-hybrid cDNA library as previously described^17^. Briefly, for cDNA library screening, the yeast reporter strain L40 was first transfected with baits pLexA-Rab3D and subsequently transfected with the pVP16 mouse embryo cDNA library using lithium acetate and polyethylene glycol. Library plasmids were grown in the presence or absence of histidine. Positive clones isolated from the cDNA library were further analysed by co-transfection with pLexA-Rab3D and DNA sequencing.

### RT-PCR

To determine the calmodulin and Rab3D gene transcripts in osteoclasts, total RNA was isolated from bone marrow cells treated with RANKL at various time points according to the manufacturer’s instructions (Qiagen, Sydney). For RT-PCR, single-stranded cDNA was prepared from 2 μg of total RNA using reverse transcriptase with an oligo-dT primer. Two μl of each cDNA was subjected to 30 cycles of PCR (94 °C, 40 sec; 55 °C, 40 sec; and 72 °C, 40 sec) using specific primers to mouse *rab3d* gene (forward: 5′-ATG GCA TCC GCT AGT GAG-3′; reverse:5′-CTA ACA GCT GCA GCT GCT-3′) and calmodulin (forward: 5′-CCA TGG CTG ACC AGC TGA-3′; reverse: 5′-CCT TCA CTT TGC AGT CAT-3′). Osteoclast markers were also used; including cathepsin K (forward: 5′-CCA GTG GGA GCT ATG GAA GA-3′; reverse: 5′-AAG TGG TTC ATG GCC AGT TC-3′); calcitonin receptor (forward: 5′-CGG ACT TTG ACA CAG CAG AA-3′, reverse: 5′-CAG CAA TCG ACA AGG AGT GA-3′); V-ATPase-d2 (forward: 5′-GTG AGA CCT TGG AAG ACC TGA A-3′; reverse:5′-GAG AAA TGT GCT CAG GGG CT-3′). As an internal control, the single stranded cDNA was PCR-amplified for 25 cycles using 36 B4 primers (forward: 5′-TCA TTG TGG GAG CAG ACA-3′; reverse: 5′-TCC TCC GAC TCT TCC TTT-3′).

### *In vitro* protein interaction

The full length of mouse Rab3D cDNA was subcloned into Flag-tagged expression vector with CMV promoter to generate a Flag-Rab3D plasmid. To express Rab3D protein, 5 × 10^5^ COS-7 cells was transfected with 10 μg of Flag-Rab3D plasmid using effectant reagents (Qiagen, Sydney). After incubation for 48 hours, transfected cells were washed twice with PBS and lysed for 30 mins on ice with 750 μl of lysis buffer (50 mM Tris.Cl, pH 7.5, 150 mM NaCl, 0.1% Nonidet P-40, 1 M EDTA, 1 μg/ml pepstatin A). Following centrifugation at 12,000 RPM for 5 mins at 4 °C, supernatant was collected and 750 μl of binding buffer (20 mM Tris. Cl, pH 7.5, 50 mM KCl, 100 mM NaCl, 2 mM CaCl_2_, 2 mM MgCl_2_, 5 mM DTT) was added to the supernatant and mixed. One half of the sample was added to 50 μl of calmodulin immobilized in sepharose beads (Sigma, Sydney) in the presence and absence of 2 mM of calcium. The mixture was incubated overnight at 4 °C with a shaker. The beads were washed three times with 300 μl of buffer containing equal volumes of lysis buffer and binding buffer. The bound proteins were boiled with 1 x SDS-PAGE sample buffer and analysed by Western blot analysis using an anti-FLAG monoclonal antibody (Sigma, Sydney).

### Construction of EYFP-Rab3D and Rluc-calmodulin

EYFP-Rab3DWT, EYFP-Rab3DQ81L, EYFP-Rab3DN135I, and EYFP-Rab3DΔCXC fusion constructs were reported previously^17^. To generate Rluc-calmodulin construct, calmodulin mRNA was PCR-amplified with primers (forward: 5′-AAG AAT TCG GAC CAT GGC TGA CCA GCT GA-3′; reverse: 5′-GGA TCC TCT AGA CCT TCA CTT TGC AGT CAT-3′). The amplified fragment was cloned into a pCR 2.1 T/A-cloning vector as outlined by the manufacturers’ instructions. The *Bam*HI and *Eco*RI fragment was then cloned into the *Bam*HI and *Eco*RI sites of pcDNA3.1-Rluc (Invitrogen) to make pcDNA-Rluc as previously described^40^. To generate a Rluc-calmodulin calcium insensitive mutant, pcDNA3.1 calcium insensitive calmodulin mutant, in which the first Asp of each EF-hand is changed to Ala, was obtained from Dr. Blaise Z. Peterson^18^ and then subcloned into the pcDNA3.1-Rluc. These constructs were sequenced to confirm. Bioluminescence resonance energy transfer (BRET) assay and cell transfections were performed in COS cells as previously described using the Mithras LB940 BRET plate reader (Berthold Technologies, Inc., Germany)^40^.

### Generation and isolation of osteoclastic cells and bone resorption assay

OCs were generated from mouse BMMs treated with RANKL (100 ng/ml) and M-CSF (10 ng/ml) in *vitro* as previously described^41^. Bone resorption assay was carried out using mouse BMM-derived mature osteoclasts as previously described^17^.

### Sucrose gradient ultracentrifugation

Sucrose gradient centrifugation was performed as described previously with minor modifications. Briefly, ~3 × 10^7^ OCs were pelleted, 500 μl of the clarified supernatant was layered on top of 12 ml of 5–20% linear sucrose density gradient prepared in the lysis buffer without Triton X-100. After centrifugation at 150,000 *g* for 18 h in a SW40 rotor (Beckman Coulter), 1 ml fractions were collected and analyzed by immunoblotting^17^ using antibodies to GFP (Santa Cruz, CA, USA), Rab3D (SynapicSystems, Goettingen), V-ATPase d2^42^ and calmodulin (Sigma, Sydney).

### Immunofluorescence confocal microscopy

Immunofluorescence detection of Rab3D in osteoclasts by confocal microscopy was performed as previously described^16^. Briefly BMM-derived osteoclasts cultured on bone for 7-days where fixed with 4% paraformaldehyde, permeablised with Triton X-100, blocked in 0.2% BSA-PBS, immunostained with primary antibodies against Rab3D (Rabbit polyclonal; SynapicSystems, Goettingen) or Calmodulin (mouse monoclonal, Sigma, Sydney) and then corresponding secondary Alexa-Fluor conjugated antibodies against rabbit (Alexafluor-488) and mouse Alexafluor-555). F-actin rings where detected by staining with Alexfluor647-conjugated Phalloidin (Molecular Probes, USA) and nuclei were visualised by staining with DAPI (Sigma, USA).

### Statistics

The results are representative of at least three independent experiments. Single comparison tests were performed by using paired Student’s t-test in Microsoft Excel. All data are presented as the mean ± standard error of the mean (SEM). Statistical significance was determined at P values < 0.05.

## Additional Information

**How to cite this article**: Zhu, S. *et al.* Calmodulin interacts with Rab3D and modulates osteoclastic bone resorption. *Sci. Rep.* **6**, 37963; doi: 10.1038/srep37963 (2016).

**Publisher's note:** Springer Nature remains neutral with regard to jurisdictional claims in published maps and institutional affiliations.
